# Characteristics of three lower limb joint kinetics affecting rebound jump performance

**DOI:** 10.1371/journal.pone.0268339

**Published:** 2022-08-15

**Authors:** Amane Zushi, Takuya Yoshida, Kodayu Zushi, Yasushi Kariyama, Mitsugi Ogata

**Affiliations:** 1 Japan Institute of Sports Sciences, Tokyo, Japan; 2 Faculty of Health and Sport Sciences, University of Tsukuba, Ibaraki, Japan; 3 Faculty of Economics, Shiga University, Shiga, Japan; 4 Faculty of Sport Sciences, Yamanashi Gakuin University, Yamanashi, Japan; Universita degli Studi di Milano, ITALY

## Abstract

The stretch-shortening cycle (SSC) motor execution ability of the lower limb was measured using the rebound jump index (RJ-index) in RJ test; this performance is influenced by the interaction of the forces exerted by the three joints of the lower limb.We aimed to determine RJ performance variables and identify the lower limb kinetic variables that affect them. One hundred two female university students (age, 20.1±1.0 years; height, 164.6±7.2 cm; mass, 58.9±7.3 kg) for whom RJ performance variables (RJ-index, jump height, and contact time) and joint kinetics (torque, power, and work) were measured. Statistical analysis showed a strong correlation between the RJ-index and jump height or contact time (r = 0.920, -0.726, p < 0.05) but a weak correlation between the jump height and contact time (r = -0.384, p < 0.05). Furthermore, positive ankle power was the most influential factor for RJ performance variables; additionally, positive knee power and hip work and eccentric knee torque significantly influenced jump height, and positive ankle power, negative work and power, and concentric torque significantly influenced the contact time. The acquisition of the jump height and a shorter contact time requires different kinetic variables. Furthermore, the characteristics of the force exerted by the three joints of the lower limb that compose the RJ-index may be different even if the RJ-index has the same value. Therefore, by assessing not only the RJ-index but also the jump height, contact time, and characteristics of lower limb joint kinetics in the RJ test, it is possible to conduct effective training to improve lower limb SSC motor execution performance according to individual characteristics.

## Introduction

The stretch-shortening cycle (SSC) movements of the lower limbs are performed during running, jumping, and change of direction in athletics and ball games [1]. Therefore, athletes can enhance their performance in competitions by improving their ability to execute SSC movements in the lower limbs during short contact times. The SSC ability of the lower limbs is assessed using the reactive strength index (RSI) [2] or rebound jump index (RJ-index) [3, 4] of the drop jump (DJ) or rebound jump (RJ) [4], which are vertical jumping motions performed with both legs. Both abovementioned indices can be obtained by dividing the jump height by the contact time during the jump. This index is widely used in research and assessment of athletes because it can be measured with a high accuracy [5–9].

Athletes with high athletic performance have higher performance variables, such as the RJ-index [4, 10]. Especially, it has been reported that the RJ-index are related to athletic activities, such as sprinting [10–14], change of direction [15, 16]. Thus, the significance of increasing the RSI and RJ-index has been shown; however, methods to improve these indices by training are not well-known [5, 9, 17]. In other words, there is a lack of knowledge on how to improve SSC motor execution ability in the lower limbs. Additionally, because the performance variables in the DJ and RJ are the result of the interrelationship of the forces exerted by the muscles and tendons of the three lower limb joints (ankle, knee, and hip), an assessment of the lower limb joint kinetics is useful for a more detailed evaluation of the athlete’s SSC ability [14, 18]. To date, there is only one study that has reported the characteristics of lower limb joint kinetics during RJ. In this study, the ankle joint torque during the eccentric phase of takeoff was related to the jump height and contact time in the RJ test [19]; however, the effects of and relationship to the other joints were not considered. Previous studies have reported that the group with a higher RJ-index has a higher ground reaction force and power in the eccentric phase [9, 20], but the effects of a higher RJ-index on the kinetic variables of the three lower limb joints have not been shown.

Furthermore, the relationship between the jump height and contact time is weak in DJ [18], and the characteristics of lower limb joint kinetic variables differ depending on the jumping technique [21]. However, the degree of influence on the performance variables has not been clarified. Therefore, in addition to the characteristics of the RJ-index, jump height, and contact time, presenting the kinetic variables of the three joints of the lower limb joints that affect the performance variables will provide knowledge for evaluating the SSC ability of the lower limbs with the RJ-index and using the results for training.

The purpose of this study was to clarify the lower limb joint kinetics that affected the RJ-index and its components, such as the jump height and contact time. We hypothesized that among the various parameters, the ankle joint power would have the greatest effect on jumping exercises performed during a task as short and high as RJ. In addition, we hypothesized that the relationship between the jump height and contact time of the RJ reflected an independent ability, such that the lower limb kinetics that affected each of these abilities were different.

## Methods

### Participants

In total, 102 female university athletes (age, 20.1±1.0 years; height, 164.6±7.2 cm; mass, 58.9±7.3 kg; breakdown of participants per event: soccer = 26, basketball = 20, handball = 23, and track and field = 33) were enrolled in this study. All subjects were members of the university sports club, competed from the regional to the international level in the preceding year, and trained with their teams approximately five days a week. All participants were recruited as volunteers from undergraduate and graduate students belonging to athletic clubs; therefore, no participant was paid. They were familiar with the experimental trials. The exclusion criteria were the use of medications affecting the exercise capacity, orthopedic limitations, or a history of a major injury. The participants in this study had experience with strength and plyometric training (including the RJ). During the study period, the subjects followed a normal training program as it was the middle of the practice season, and they did not engage in any strenuous exercise the day before the measurements were performed. There were no injuries to the lower limbs that could have affected their ability to perform RJs. This study was approved by the Research Ethics Committee of the affiliations, and the subjects were informed of the benefits and risks of the investigations prior to signing an institutionally approved informed consent document to participate in the study (tai 30–142).

### Experimental protocol

The measurement period was from 2019 to 2020, and all measurements were performed in a muscle strength and power measurement room. Participants were instructed to place their　each feet separately on two force plates (Kistler 9287C, Kistler Instrumente AG, Winterthur, Switzerland). All subjects performed the RJ at least three times as a warm-up. The RJ involved repeated jumps and was performed with six jumps [10, 19]. The participants were instructed to jump as high as possible and keep the contact time as short as possible. Before conducting the experiment, the participants practiced the protocol to ensure that the experiment could be performed correctly, and the author, who was familiar with the experimental protocol, instructed the participants accordingly. To avoid the possibility of bias from instructions, no instructions were given during the measurements. The jump with the highest RJ-index was chosen for the statistical analysis. If the ground contact of the jump was outside the force plate, it was considered a failed attempt. To measure the two force plates when stepping on each of the left and right feet, the measurer visually checked the task, and a video was recorded to confirm that the stepping was performed correctly. Adequate rest time (approximately 120 seconds) was allowed between trials to minimize the effects of fatigue as much as possible.

### Data collection and processing

The three-dimensional coordinates of 13 retro-reflective markers (diameter: 14 mm) fixed on the body were collected using a Vicon T20 system, with 10 cameras operating at 250 Hz. Auto-labeling was used for the measurement of body coordinates in several people. In the preliminary experiment, it was expected that the coordinates of the left and right hip joint markers would be swapped during the measurement; hence, a dummy marker was attached to one thigh to deal with the problem. If swapping of the coordinates of the hip joint marker occurred during the trial, the swapped coordinates were re-labeled. The reflective marker was fixed around the periphery with kinesiology tape (NITREAT Kinesiology Tape, Nitto Group Company, Osaka, Japan) so that it would not come off during the measurement.

The Ground reaction Force (GRF) was measured using two force platforms (Kistler 9287C, 0.9 m × 0.6 m, Kistler Instrumente AG, Winterthur, Switzerland) at 1,000 Hz. The kinematic data were smoothed using a fourth-order, low-pass Butterworth filter with optimal cut-off frequencies of 7.5 and 15.0 Hz. These data were time-synchronized using Vicon Nexus software (Nexus 2, Vicon Motion Systems, Ltd., Oxford, UK) for subsequent inverse dynamic analysis. The kinetic measurements of the dominant leg were used for the data analysis.

The ground contact and air times were calculated at the point where the vertical GRF became <10 N. The jump height was calculated using the following free-fall formula: jump height = (g ^1^ t_air_^^2^^) 8⁻^1^, with ‘g’ as the gravitational acceleration with a value of 9.81 m/s^2^. The RJ-index was calculated by dividing the jump height by the contact time [2, 4].

**Fig 1:**
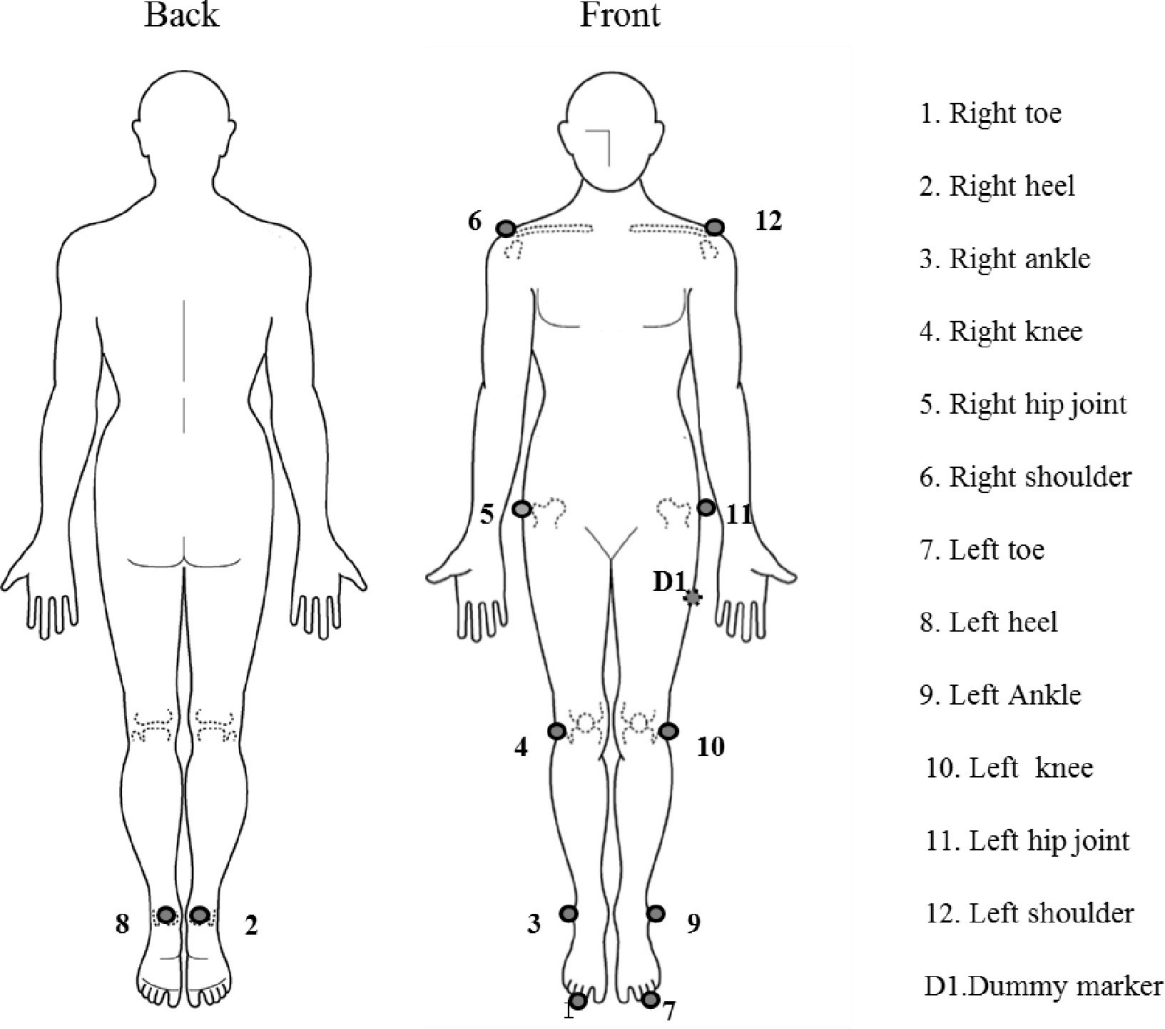
Reflection marker position.

**Fig 2:**
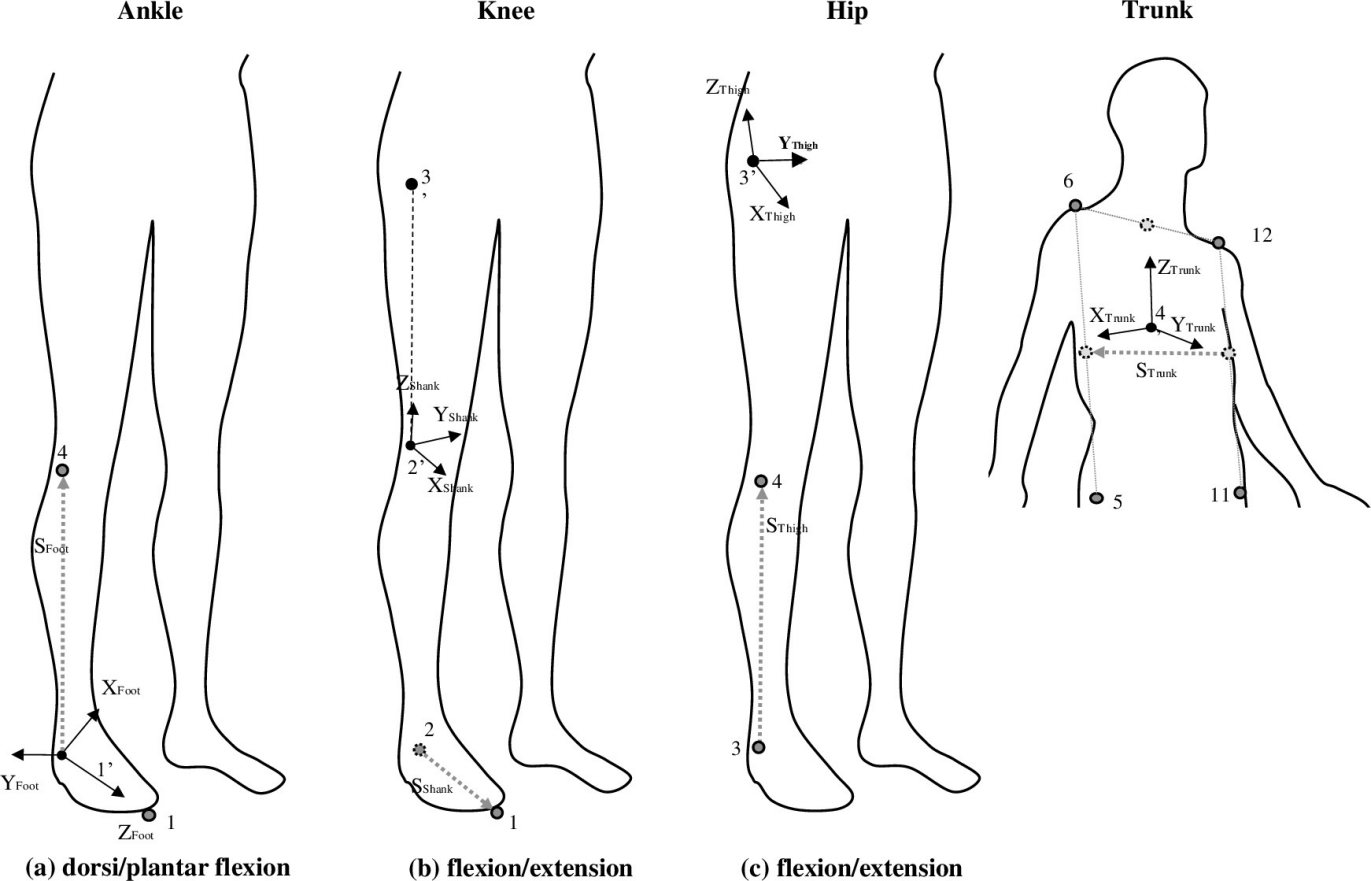
Coordinate system fixed at the hip, knee, and ankle joints to calculate each joint angle. 1: Right toe, 2: Right heel, 3: Right ankle, 4: Right knee, 5: Right hip joint, 6 Right shoulder, 11: Left hip joint, 12: Left shoulder.

Twelve representative body points (Fig 1) were used in each subject (1: right toe, 2: right heel, 3: right ankle, 4: right knee, 5: right greater trochanter, 6: right shoulder, 7: left toe, 8: left heel, 9: left ankle, 10: left knee, 11: left greater trochanter, and 12: left shoulder), and one dummy marker was attached to the left thigh. The coordinate system used to calculate the joint torque and joint angle was the vector from the ankle joint to the toe is the Z-ankle axis, the vector from the knee joint to the ankle joint is the auxiliary vector S-ankle axis, and the outer product of the Z-ankle and S-ankle axes is the Y-ankle axis. The outer product of the Y-ankle and Z-ankle axes is the X-ankle axis, and the moving coordinate system consisting of the X-ankle, Y-ankle, and Z-ankle axes is termed as the ankle joint moving coordinate system. The vector from the knee joint to the ankle joint is the Z-knee axis, the vector from the greater trochanter to the knee joint is the auxiliary vector S-knee axis, and the outer product of the Z-knee and S-knee axes is the Y-knee axis. The outer product of the Y-knee and Z-knee axes is the X-knee axis, and the moving coordinate system consisting of the X-knee, Y-knee, and Z-knee axes is the knee joint moving coordinate system. The vector from the hip joint to the knee joint is the Z-hip axis, the vector from the knee joint to the ankle joint is the auxiliary vector S-hip axis, and the outer product of the Z-hip and S-hip axes is the Y-hip axis. The outer product of the Y-hip and Z-hip axes is the X-hip axis, and the moving coordinate system consisting of the X-hip, Y-hip, and Z-hip axes is the hip joint moving coordinate system. The vector that goes from the center of gravity to the midpoint of the left and right shoulders is the Z-trunk axis, and the vector that goes from the midpoint of the left abductor and left shoulder to the midpoint of the right abductor and right shoulder is the auxiliary vector S-trunk axis. The outer product of the Z-trunk and S-trunk axes is the Y-trunk axis. The outer product of the Y-trunk and Z-trunk axes is the X-trunk axis, and the moving coordinate system consisting of the X-trunk, Y-trunk, and Z-trunk axes is the trunk moving coordinate system (Fig 2) [22].

The coordinates were smoothed by means of a fourth-order, zero-lag, low-pass Butterworth filter with optimal cut-off frequencies of 7.5–15 Hz, which were determined using the residual method [23]. The center of mass and the inertial parameters were estimated based on the body-segment parameters of Japanese athletes [24]. The global coordinate system was defined using the participants’ jumping directions in the X (mediolateral direction), Y (anterior-posterior direction), and Z (vertical direction) axes. The ankle joints were analyzed for plantarflexion and dorsiflexion, and the knee and hip joints were analyzed for extension and flexion. The ankle joint angle was defined as the line segment connecting the ankle and knee and that connecting the ankle and toe, the knee joint angle was defined as the line segment connecting the knee and greater trochanter and that connecting the knee and ankle, and the hip joint angle was defined as the angle formed by the line segment connecting the greater trochanter and shoulder and that connecting the greater trochanter and knee. Angular velocity was calculated as the average of the negative and positive values for each joint, considering the flexion velocity with the negative value and the extension velocity with the positive value.

Joint kinetics was divided into the first and second half of the takeoff based on the lowest point of the center of gravity. Joint torque was calculated using an inverse dynamics approach. The joint torque at each joint was transformed into the joint coordinate system (Fig 1). The joint power was computed as the dot product of the joint torque and joint angular velocity, and the average values of the negative and positive power due to the extension torque during the takeoff phase were calculated. The negative joint work and positive joint work were calculated by integrating the power over time. These data were determined around the positive extension-negative flexion (positive plantarflexion-negative dorsiflexion) axis around the ankle, knee, and hip joints [22].

### Statistical analysis

Intraclass correlation coefficients (ICCs) were calculated to determine the inter-measurement reliability of the measured variables. The reliability of the RJ-index in the analyzed data was also examined using the Shapiro-Wilk test. The Pearson’s product rate correlation coefficient was used to analyze the correlation between the RJ-index, jump height, and contact time. To examine the effects of the kinetic variables (torque, power, and work) of the three lower limb joints on the RJ-index, jump height, and contact time, a multiple regression analysis using the stepwise method was performed with the RJ-index, jump height, and contact time as the dependent variables and the kinetic variables of the three lower limb joints as the independent variables. For the determination of these parameters, we referred to the kinetic variables of the lower limb joints that were utilized in previous studies [19, 22, 25]. To examine the influence of the ankle, knee, and hip joints on the RJ-index, jump height, and contact time, multiple regression coefficients were calculated and a Pearson correlation analysis was performed by standardizing each variable to a mean of 0 and a variance of 1. The alpha level was set at 0.05. All data are presented as mean ± standard deviation (SD). Statistical analyses were performed using SPSS (version 25, IBM Corp., NY, USA).

## Results

The inter-measurement reliability of the RJ test in this study was high (ICC: RJ-index = 0.948, jump height = 0.947, contact time = 0.850). The reliability of the RJ-index was examined using the Shapiro−Wilk test, with a statistic of 0.980.

**Fig 3:**
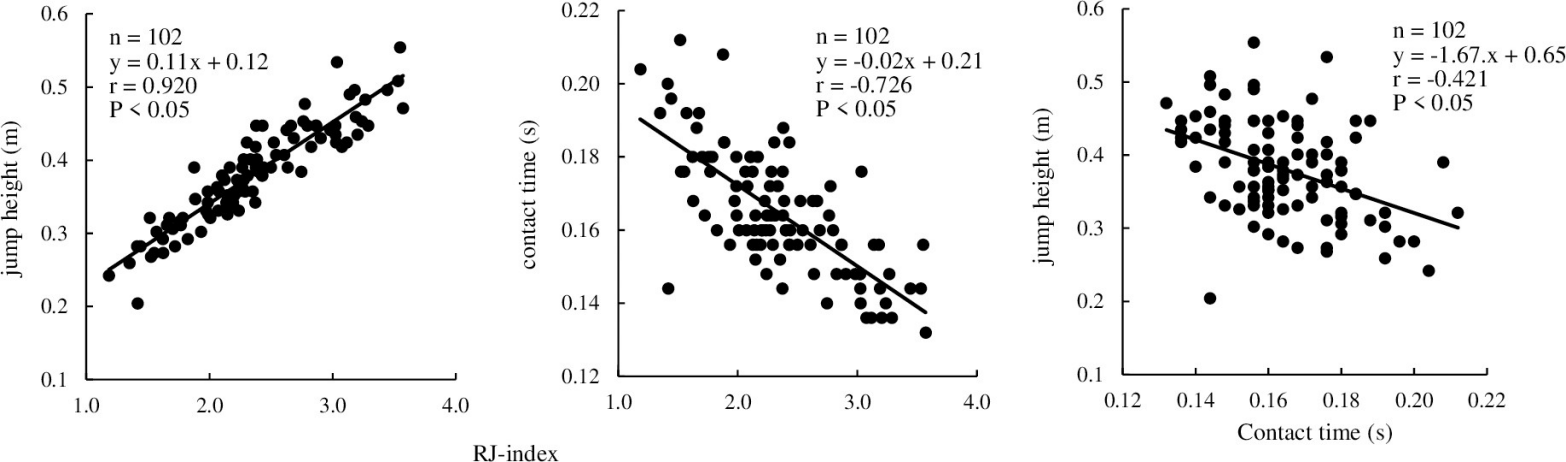
Relationship among performance variables in the rebound jump test.

Table 1 shows the calculated variables in this study as mean ± SD. Fig 3 shows the correlation among the performance variables of RJ. A significantly high correlation was found between the RJ-index and contact time and between the RJ-index and jump height, while a moderate correlation was found between the contact time and jump height (r = 0.920, -0.726, -0.421, p < 0.01).

**Table 1:** Parameters of rebound jump performance.

Parameters		mean	±	SD	Min	Max
**Performance variables**						
	RJ-index	2.35	±	0.55	1.19	3.57
	Jump height(m)	0.38	±	0.07	0.20	0.55
	Contact time (s)	0.16	±	0.02	0.13	0.21
**Max torque (Nm/kg)**						
	Hip	3.16	±	1.15	0.52	7.14
	Knee	4.15	±	1.10	2.01	7.61
	Ankle	4.42	±	0.76	2.94	6.97
**Eccentric torque (Nm/kg)**						
	Hip	1.95	±	1.02	0.06	5.61
	Knee	2.41	±	0.63	0.88	4.31
	Ankle	2.72	±	0.47	1.76	4.33
**Concentric torque (Nm/kg)**						
	Hip	1.44	±	0.96	0.21	5.17
	Knee	2.01	±	0.37	1.07	3.04
	Ankle	2.40	±	0.36	1.57	4.04
**Negative power (W/kg)**						
	Hip	-3.31	±	2.74	-0.03	-15.28
	Knee	-8.96	±	3.47	-2.56	-21.03
	Ankle	-19.95	±	5.92	-9.97	-38.26
**positive power (W/kg)**						
	Hip	3.87	±	1.81	0.42	8.78
	Knee	11.75	±	3.68	4.28	24.24
	Ankle	15.57	±	3.68	8.75	27.19
**Negative work(J/kg)**						
	Hip	-0.17	±	0.13	-0.01	-0.59
	Knee	-0.61	±	0.27	-0.08	-1.50
	Ankle	-1.20	±	0.25	-0.68	-2.12
**Positive work (J/kg)**						
	Hip	0.37	±	0.20	0.03	0.96
	Knee	0.78	±	0.22	0.31	1.54
	Ankle	1.49	±	0.29	0.93	2.67

Table 2 shows the results of the multiple regression analysis with the RJ-index as the dependent variable and lower limb kinetics data as the explanatory variables, which are as follows: positive power (β = 0.809, p < 0.00), negative power (β = -0.589, p < 0.00), negative work (β = 0.410, p < 0.00), and concentric torque (β = -0.267, p < 0.00). Positive power at the knee (β = 0.242, p < 0.00), positive work at the hip joint (β = 0.228, p < 0.00), and eccentric torque at the knee (β = 0.172, p < 0.00) were extracted as significant factors.

**Table 2:** Multiple regression predictors of the rebound jump index.

		Multiple regression analysis		
		β	t	p value
1	**Ankle positive power (W/kg)**	0.809	8.780	0.000
2	**Ankle negative power (W/kg)**	-0.589	-6.459	0.008
3	**Ankle negative work (J/kg)**	0.410	4.540	0.000
4	**Ankle concentric torque (Nm/kg)**	-0.267	-2.724	0.003
5	**Knee positive power (W/kg)**	0.242	4.805	0.000
6	**Hip positive work** **(J/kg)**	0.228	4.393	0.000
7	**Knee eccentric torque (Nm/kg)**	0.172	3.008	0.000

Statistically significant difference: p < 0.05

Multiple regression analysis was performed using the factors extracted as influencing the RJ-index, with the jump height and contact time as dependent variables and lower limb joint kinetic variables as explanatory variables. First, positive power of the ankle (β = 0.359, p < 0.00), positive power of the knee (β = 0.283, p < 0.00), positive work of the hip (β = 0.265, p < 0.00), and eccentric torque of the knee (β = 0.201, p < 0.00) were extracted as significant factors for jump height (Table 3). Next, positive power (β = -0.954, p < 0.00), negative work (β = -0.890, p < 0.00), negative power (β = 0.885, p < 0.00) and concentric torque (β = 0.325, p < 0.00) of the ankle were extracted as significant factors for the contact time (Table 4).

**Table 3:** Multiple regression predictors of jump height.

		Multiple regression analysis		
		β	t	p value
1	**Ankle positive power** **(W/kg)**	0.359	4.200	0.000
2	**Knee positive power** **(W/kg)**	0.283	4.395	0.000
3	**Hip positive work** **(J/kg)**	0.265	4.200	0.000
4	**Knee eccentric torque (Nm/kg)**	0.201	2.784	0.006

Statistically significant difference: p < 0.05

**Table 4:** Multiple regression analysis of predictors of contact time.

		Multiple regression analysis		
		β	t	p value
1	**Ankle positive power** **(W/kg)**	-0.954	-10.514	0.000
2	**Ankle negative work (J/kg)**	-0.890	-10.140	0.000
3	**Ankle negative power** **(W/kg)**	0.885	10.776	0.000
4	**Ankle concentric torque (Nm/kg)**	0.325	3.436	0.001

Statistically significant difference: p < 0.05

## Discussion

The results of this study supported the hypothesis that, among the various parameters, variables related to the ankle joint would have the greatest influence on the RJ-index. In addition, we hypothesized that the relationship between the jump height and contact time of the RJ reflected an independent ability and the lower limb kinetics that affected each of these abilities were different. The results of this study supported these hypotheses because it was found that joint kinetics related to the knee and hip joints, but not the ankle joint, had different effects on jump height and contact time.

The RJ-index showed high reliability (ICC: 0.948). The characteristics of the kinetic variables of the three lower limb joints in the RJ test were evaluated. Table 1 shows that the ankle joint had the highest values for all parameters, followed by the knee and hip joints. Second, multiple regression analysis was performed to examine the effect of the torque, power, and work of the three lower limb joints on the RJ-index. The results revealed that the positive power, negative power, negative work, and concentric torque of the ankle joint, the positive power of the knee joint, the positive work of the hip joint, and the eccentric torque of the knee joint affected the RJ-index, in that order (Table 2). These results show that the contribution of the ankle joint is greater among the three joints of the lower extremities in jumping exercises performed with instructions to jump as high as possible with a short ground contact time, such as in the RJ [21, 22]. Furthermore, because of the constraint of keeping the ground contact time as short as possible, the parameter of power, calculated as the product of torque and time, was considered to have the greatest influence.

Using the parameters that affected the RJ-index, the magnitude of the effect of each of these parameters was examined separately for the jump height and contact time. The results showed that the jump height was highly affected by the positive ankle joint force, followed by the positive knee joint force, positive hip joint work, and eccentric knee joint torque (Table 3). Contrarily, the contact time was highly affected by the positive force, followed by the negative work, negative force, and concentric torque of the ankle joint (Table 4). These results indicated that the positive ankle power was the most influential parameter on a series of performance variables, such as the jump height, contact time, and RJ-index (Tables 2–4). The muscle-tendon complex involved in ankle plantarflexion has been shown to be excellent at storing large amounts of elastic energy when stretched for short periods and then reusing that energy during the concentric phase to enhance force exertion [26]. In addition, it has been shown that there is a correlation between ankle joint kinetics variables and the contact time during the takeoff phase of the RJ [4], and it is thought that the ankle extensor muscles can exert a greater force in a shorter time, leading to a shorter contact time. Moreover, it has been shown that the magnitude of the force exerted by the ankle plantar flexors at the beginning of shortening is also advantageous for the acquisition of the jump height [19]. Furthermore, the acceleration of the center of gravity of the body necessary to obtain the jump height is produced by the power output of the involved muscle groups around the knee and ankle joints during takeoff, and the bounce-type DJ with a short contact time has a higher power output at the knee and ankle joints than does the countermovement jump or the countermovement-type DJ with a long contact time [25]. As power is calculated using torque and time, the concentric torque of the ankle joint was considered to be a factor affecting the RJ-index and contact time, because the calculation of the ankle joint torque does not take displacement into account. These results suggest that the positive power of the ankle joint affects the acquisition of the jump height and the reduction of the contact time and is the most influential parameter for the acquisition of a high RJ-index. Although this is only indicative of the fact that the torque of the ankle joint was related to the jump height and contact time of the RJ, the results of this study suggested that, among the variables of torque, power, and work, the positive power of the ankle joint had the maximum influence on the acquisition of the jump height, contact time, and a high RJ-index.

A weak correlation was observed between the contact time and jump height, components of the RJ-index (r = -0.384, P < 0.05) (Fig 3). Previous studies have shown that there is no correlation between the jump height and contact time and that they are independent of each other [18, 27, 28]. Therefore, the RJ-index is composed of two independent components, suggesting that there may be cases with the same RJ-index that have characteristics such as a high jump height but a long contact time or a short contact time but a low jump height. This indicates that several subjects cannot achieve both a high jump height and shortened contact time, which are both factors that affect the RJ-index. Previous studies using the DJ have shown that there is no correlation between the jump height and contact time and that they are independent of each other [4, 18, 27]. Thus, our findings related to the RJ components are in agreement with the findings in these previous studies.

The eccentric torque and positive power of the knee joint were shown to affect the jump height (Table 3). It has been shown that a high knee joint stiffness after ground contact plays a key role as a power source in regulating DJ performance by increasing the rate of the center-of-gravity elevation, which is acheived by influencing the facilitation of the stretch reflex [29, 30]. Thus, it is possible that the knee joints are compensating for the load that the ankle joint could not take in order to receive a larger force. In other words, the ankle and knee joints convert the larger force received into a force used to obtain a higher jump height. However, a greater amount of knee joint flexion immediately after landing leads to an increase in the contact time [31]. Therefore, the eccentric knee torque, which is not related to displacement, was extracted as an influential parameter. It is suggested that the knee joint is related to receiving the body weight by exerting eccentric force with as little flexion as possible in the eccentric phase of takeoff and obtaining a high jump height by exerting a large amount of power in the concentric phase of takeoff.

In addition, the positive work of the hip joint was extracted for the jump height (Table 3). Compared with the ankle and knee joints, the hip joint is characterized by a large surface area of intervening muscle groups; this is excellent for exerting a large force but disadvantageous for exerting a quick force due to its large moment of inertia. Moreover, the hip joint has structural and functional characteristics that enable it to exert a large amount of force because of the large area of intervening muscle groups, such as the gluteus maximus, hamstrings, and adductor muscles. Furthermore, the hip joint is located in the center of the body, which is an in-line articulated system, and the force and power exerted there are transmitted to the knee and ankle joints through interarticular forces and biarticular muscles [32]. Thus, the hip joint is responsible for generating energy to obtain the jump height, and the hip extensor muscle group is the most significant force exerted by the hip joint not only in DJs that enable a person to jump as high as possible without considering ground contact time, as in countermovement drop jump [25], but also in exercises that require the person to jump as high as possible in as short a time as possible, as in RJ. However, considering that the relationship was found only for positive hip work and not for other parameters, including negative work, it is possible that the jumping motion may be influenced by the hip joint’s ability to exert positive work even for a short ground contact time. Therefore, the positive work of the hip joint in RJ was considered to affect only the height of the jump and not the contact time.

In contrast, for the contact time, only the parameters of the ankle joint were extracted, and positive power was followed by negative power and negative work (Table 4). Power was calculated by the inner product of the torque and angular velocity. Since the eccentric torque was not extracted as a factor affecting the RJ-index in this study, it is considered that the quicker transition to the push-off phase after ground contact affects the demonstration of large negative power. Furthermore, negative work was calculated by integrating the negative power over time. Therefore, it is considered that the larger negative power is related to the larger negative work, even during the short contact time. These results suggest that the positive and negative power of the ankle joint, which are favorable for shortening the contact time, and the larger negative work due to the larger negative power affect the shortening of the contact time.

This study has some limitations. First all the subjects were females. If the same RJ-index is achieved by male and female athletes, there will be a difference in the jump height and contact time, i.e., the results of this study may show different characteristics depending on sex. Second, because this study used cross-sectional data, future studies on longitudinal changes are necessary to further examine the parameters of the three lower limb joints that affect the improvement of the RJ-index.

## Conclusions

The results indicate that the power of the ankle joint was the most influential factor on the RJ-index, and different kinetics variables of the lower limb joints are involved in the reduction of the contact time and the acquisition of the jump height, which are components of the RJ-index. This suggests that the reduction of the contact time and the acquisition of the jump height may require different motor skills. Moreover, even if the RJ-index is the same, the ability to shorten the contact time and to increase the jump height may differ, and the characteristics of the forces exerted by the three joints of the lower limb that constitute these abilities may also differ.

## Supporting information

S1 Table Individual data of RJ performance and kinetics variables at the three lower lower joints. (PDF)
