# Supplementary material for: Characteristics of three lower limb joint kinetics affecting rebound jump performance
Source: PLoS One. 2022 Aug 15;17(8):e0268339. doi: 10.1371/journal.pone.0268339 (PMC9377628; doi:10.1371/journal.pone.0268339)
Supplement: S1 Table — (PDF) [file pone.0268339.s001.pdf]

	Rebound jump performance			Max torque (Nm/kg)			Eccentric torque (Nm/kg)			Concentric torque (Nm/kg)			Negative power (W/kg)			Positive power (W/kg)			Negative work(J/kg)			Positive work (J/kg)		
	RJ-index	contact time	jump height	ankle	knee	hip	ankle	knee	hip	ankle	knee	hip	ankle	knee	hip	ankle	knee	hip	ankle	knee	hip	ankle	knee	hip
1	2.607	0.156	0.41	4.58	3.44	4.10	3.07	2.12	2.99	2.61	1.94	1.56	-21.97	-12.47	-8.23	21.14	13.69	6.03	-1.48	-0.69	-0.36	1.77	0.82	0.59
2	2.499	0.156	0.39	4.98	4.39	3.02	2.85	2.21	3.02	2.44	2.02	2.92	-22.36	-11.18	-3.57	15.81	14.62	3.64	-1.34	-0.86	-0.18	1.51	1.00	0.28
3	2.429	0.156	0.38	4.37	5.39	2.75	2.98	2.89	1.53	2.48	2.17	0.78	-29.19	-8.74	-2.92	14.69	13.21	2.77	-1.50	-0.42	-0.09	1.53	0.79	0.25
4	2.825	0.148	0.42	4.36	3.90	5.61	2.96	2.40	5.61	2.73	1.96	5.17	-23.54	-7.98	-9.47	15.02	12.50	8.78	-1.13	-0.49	-0.37	1.50	0.70	0.86
5	2.377	0.164	0.39	5.27	3.10	3.81	3.23	1.67	3.81	2.34	1.71	3.21	-19.22	-7.46	-6.04	14.83	10.62	4.32	-1.14	-0.57	-0.28	1.54	0.56	0.46
6	2.627	0.168	0.44	4.12	3.62	2.63	2.79	2.60	2.63	2.27	2.16	2.21	-19.18	-8.72	-3.17	14.99	10.95	5.80	-1.23	-0.53	-0.19	1.56	0.79	0.43
7	3.021	0.148	0.45	4.36	5.40	2.72	2.72	1.96	2.72	2.50	1.56	2.21	-22.19	-5.20	-1.00	18.87	8.28	4.00	-1.23	-0.26	-0.06	1.59	0.43	0.36
8	3.021	0.148	0.45	4.96	4.39	2.77	3.13	3.13	2.62	2.43	2.32	2.77	-21.64	-13.00	-3.64	17.03	17.64	5.66	-1.10	-0.81	-0.19	1.57	1.00	0.39
9	2.234	0.160	0.36	4.21	4.00	2.59	2.69	2.56	1.77	2.33	2.33	0.58	-16.57	-7.51	-3.24	14.71	15.20	2.41	-0.98	-0.60	-0.21	1.47	0.79	0.18
10	2.662	0.168	0.45	5.01	3.76	4.79	2.79	2.64	4.79	2.23	2.15	4.79	-14.33	-13.82	-11.48	14.13	13.08	6.44	-1.03	-0.79	-0.59	1.36	0.95	0.71
11	3.443	0.144	0.50	4.94	4.62	3.14	3.32	2.81	0.96	2.88	2.52	1.43	-26.14	-10.93	-0.80	21.97	18.38	5.84	-1.67	-0.70	-0.08	1.76	0.96	0.36
12	3.188	0.144	0.46	4.49	4.20	4.53	2.77	2.59	2.11	3.05	2.23	1.36	-23.57	-12.16	-4.33	19.75	19.64	5.17	-1.20	-0.84	-0.26	1.82	0.94	0.28
13	3.116	0.136	0.42	3.89	3.63	2.46	2.54	2.08	0.68	2.43	2.37	0.90	-19.60	-8.04	0.27	18.49	12.00	2.83	-1.08	-0.36	0.00	1.48	0.63	0.33
14	3.530	0.144	0.51	4.63	4.97	3.37	2.80	3.55	2.33	2.89	2.63	0.87	-28.19	-12.18	-5.29	18.53	21.20	2.99	-1.34	-0.79	-0.22	1.78	1.01	0.29
15	2.685	0.160	0.43	4.36	4.71	3.13	2.86	2.94	1.45	2.47	1.96	1.07	-24.30	-10.26	-3.35	15.08	14.58	3.11	-1.45	-0.74	-0.12	1.50	0.88	0.36
16	3.548	0.156	0.55	4.13	5.32	3.90	2.53	3.71	1.81	2.59	2.92	0.62	-28.30	-21.03	-5.22	16.51	24.24	4.12	-1.25	-1.50	-0.34	1.85	1.54	0.31
17	2.542	0.160	0.41	4.85	3.34	3.62	2.91	1.82	2.35	2.84	1.90	1.02	-22.57	-7.01	-3.95	21.55	13.92	4.41	-1.43	-0.56	-0.25	2.07	0.73	0.38
18	2.746	0.140	0.38	4.29	5.34	2.48	2.92	2.93	1.40	2.38	2.01	0.85	-23.97	-9.53	-5.79	18.51	12.85	2.71	-1.13	-0.47	-0.21	1.63	0.78	0.20
19	3.570	0.132	0.47	5.20	5.99	4.75	3.22	4.01	4.00	3.04	2.17	4.75	-26.52	-3.91	-3.44	27.19	6.35	3.90	-1.47	-0.22	-0.09	1.95	0.38	0.40
20	3.266	0.148	0.48	5.09	5.18	4.37	3.47	3.18	1.65	2.78	2.55	1.24	-31.62	-8.86	-0.39	20.90	15.54	3.62	-1.73	-0.50	-0.05	1.84	0.87	0.41
21	2.982	0.148	0.44	4.11	3.31	3.99	2.67	1.78	2.14	2.50	1.99	1.30	-22.68	-11.45	-7.48	18.21	13.14	6.70	-1.17	-0.72	-0.39	1.68	0.63	0.56
22	3.027	0.140	0.42	5.10	3.66	2.87	2.93	2.69	1.55	3.02	2.03	1.07	-31.28	-8.37	-2.33	21.39	14.58	3.47	-1.49	-0.46	-0.11	1.88	0.76	0.24
23	3.034	0.176	0.53	4.73	4.45	2.93	3.19	2.58	1.97	2.88	2.65	1.36	-27.00	-10.14	-0.42	19.27	15.96	3.05	-1.93	-0.87	-0.03	2.00	1.09	0.41
24	2.429	0.156	0.38	4.68	4.51	4.41	2.50	1.73	4.41	2.32	1.32	2.93	-17.02	-9.47	-5.50	17.91	9.52	3.38	-1.15	-0.63	-0.25	1.43	0.60	0.34
25	3.288	0.136	0.45	5.78	7.61	2.72	3.79	3.01	2.72	2.46	2.24	1.28	-34.25	-9.13	-2.68	19.77	12.51	2.49	-1.46	-0.44	-0.13	1.75	0.90	0.16
26	2.373	0.144	0.34	4.21	3.46	1.92	2.37	2.24	1.92	2.12	1.46	1.86	-20.21	-5.31	-0.73	15.62	8.52	1.90	-1.04	-0.28	-0.04	1.37	0.72	0.11
27	2.903	0.148	0.43	4.20	3.54	3.01	3.22	2.26	1.17	2.70	1.95	3.01	-22.08	-5.26	-0.56	22.62	10.60	3.16	-1.41	-0.35	-0.01	1.81	0.63	0.30
28	3.074	0.136	0.42	5.70	6.38	3.71	2.98	2.67	3.71	2.55	1.89	3.63	-26.11	-8.12	-5.94	18.34	14.70	6.29	-1.22	-0.48	-0.25	1.54	0.76	0.57
29	3.024	0.144	0.44	6.49	4.71	2.76	3.76	2.14	1.97	3.00	2.16	2.76	-28.66	-9.60	-4.05	27.10	13.06	4.84	-1.94	-0.54	-0.08	2.06	0.78	0.36
30	2.277	0.164	0.37	5.84	3.46	2.76	3.12	1.94	2.53	2.91	1.81	2.76	-27.30	-8.82	-5.42	16.45	12.71	3.74	-1.40	-0.72	-0.29	1.78	0.77	0.37
31	3.178	0.156	0.50	4.46	3.91	3.85	3.31	2.54	2.40	2.93	2.28	0.75	-25.15	-16.03	-8.98	18.08	15.19	4.28	-1.28	-0.96	-0.40	1.88	1.04	0.35
32	3.237	0.140	0.45	4.39	4.39	2.55	3.06	2.97	1.48	2.41	2.45	2.55	-26.30	-7.91	-1.12	16.93	13.62	4.09	-1.46	-0.44	-0.04	1.36	0.92	0.47
33	3.202	0.136	0.44	4.53	5.01	2.17	2.87	3.29	0.78	2.55	2.64	0.93	-26.09	-8.46	-0.13	17.83	13.17	3.28	-1.24	-0.39	-0.02	1.57	0.90	0.27
34	2.795	0.160	0.45	6.97	6.44	4.14	4.33	3.33	2.47	4.04	3.04	1.70	-38.26	-12.65	-3.99	26.69	17.36	5.01	-2.12	-0.77	-0.19	2.67	1.19	0.40
35	2.402	0.160	0.38	5.38	3.75	3.06	3.53	2.26	1.60	3.00	2.11	0.85	-23.06	-8.73	-1.24	20.00	11.35	2.34	-1.47	-0.58	-0.07	1.84	0.73	0.20
36	2.763	0.164	0.45	3.83	4.14	7.14	2.81	2.90	3.67	2.43	2.20	0.80	-16.86	-20.58	-15.28	15.36	13.92	2.91	-1.07	-1.14	-0.56	1.51	1.01	0.31
37	1.622	0.180	0.292	3.12	3.43	1.45	1.81	1.94	0.66	1.97	1.87	0.26	-11.26	-7.89	-1.51	9.85	11.28	1.75	-0.76	-0.69	-0.14	1.10	0.77	0.14
38	1.674	0.192	0.321	3.63	2.81	3.35	2.39	1.84	2.15	2.16	1.61	1.43	-13.76	-9.37	-5.19	15.43	10.02	4.99	-1.20	-0.61	-0.44	1.48	0.68	0.48
39	1.990	0.164	0.326	4.30	2.90	0.65	2.72	1.70	0.06	2.38	1.93	0.30	-18.75	-6.82	-0.03	18.66	7.26	0.92	-1.25	-0.46	0.00	1.64	0.55	0.04
40	2.008	0.160	0.321	5.83	4.27	2.49	3.01	2.61	0.52	2.66	1.86	0.60	-20.83	-8.97	-1.56	17.04	12.57	0.73	-1.14	-0.50	-0.08	1.67	0.65	0.07
41	2.125	0.156	0.331	5.51	3.80	3.94	3.55	2.22	3.38	2.39	1.33	1.60	-32.89	-5.53	-1.63	15.99	8.16	3.72	-1.42	-0.38	-0.04	1.67	0.69	0.35
42	2.157	0.156	0.337	4.73	4.64	1.75	2.20	3.05	0.80	2.24	2.05	0.33	-20.65	-14.56	-5.13	13.25	14.53	1.07	-0.88	-0.98	-0.11	1.45	0.92	0.08
43	2.191	0.156	0.342	6.20	6.75	5.59	2.66	2.17	2.36	2.05	2.05	1.13	-16.70	-12.56	-5.50	10.34	15.07	7.62	-0.68	-0.59	-0.25	1.15	0.83	0.47
44	2.201	0.160	0.352	4.39	3.17	2.46	2.34	2.02	1.45	2.27	1.69	0.78	-15.92	-6.12	-3.04	16.05	11.62	2.95	-1.07	-0.47	-0.16	1.47	0.55	0.25
45	2.239	0.148	0.331	4.26	3.08	2.89	2.66	1.92	2.47	2.18	1.51	1.41	-17.73	-5.76	-2.99	13.46	7.73	5.52	-0.90	-0.35	-0.10	1.19	0.50	0.55
46	2.244	0.164	0.368	4.80	5.78	3.08	3.38	2.19	0.92	2.48	1.88	1.27	-31.09	-5.58	-1.69	17.11	6.97	4.83	-1.46	-0.22	-0.07	1.65	0.64	0.47
47	2.279	0.176	0.401	5.23	2.51	2.77	2.76	1.82	1.61	2.63	1.43	1.18	-19.77	-7.32	-5.14	16.77	8.38	5.25	-1.26	-0.38	-0.28	1.81	0.74	0.56
48	2.311	0.164	0.379	6.08	2.88	4.38	3.29	1.94	2.38	2.62	1.07	1.07	-27.44	-7.07	-4.82	15.30	7.75	5.47	-1.43	-0.58	-0.36	1.70	0.50	0.39
49	2.402	0.160	0.384	4.24	4.90	3.72	2.50	1.67	1.59	2.14	1.27	1.63	-23.38	-6.48	-2.40	13.60	4.73	4.86	-1.19	-0.33	-0.08	1.30	0.34	0.58
50	1.185	0.204	0.242	2.94	2.27	1.86	1.95	1.13	1.08	1.95	1.26	0.78	-9.97	-4.87	-1.79	10.38	5.50	3.06	-0.99	-0.34	-0.14	1.08	0.44	0.34
51	1.351	0.192	0.259	3.43	3.00	1.80	2.11	1.69	1.03	1.84	1.67	0.42	-11.29	-7.31	-2.08	9.91	9.90	1.86	-0.90	-0.64	-0.17	1.03	0.72	0.16

	Rebound jump performance			Max torque (Nm/kg)			Eccentric torque (Nm/kg)			Concentric torque (Nm/kg)			Negative power (W/kg)			Positive power (W/kg)			Negative work(J/kg)			Positive work (J/kg)		
	RJ-index	contact time	jump height	ankle	knee	hip	ankle	knee	hip	ankle	knee	hip	ankle	knee	hip	ankle	knee	hip	ankle	knee	hip	ankle	knee	hip
52	1.417	0.144	0.204	3.77	2.38	1.59	2.02	0.88	0.20	1.99	1.56	0.84	-13.96	-2.56	0.32	11.60	5.21	2.04	-0.72	-0.08	-0.01	0.93	0.42	0.08
53	1.785	0.18	0.321	4.18	3.22	1.82	2.36	1.79	1.07	2.34	1.52	0.59	-19.17	-8.49	-2.83	13.64	11.24	2.80	-1.22	-0.74	-0.21	1.47	0.67	0.19
54	2.147	0.152	0.326	5.24	4.01	1.81	3.23	2.61	0.98	2.53	2.08	0.77	-21.52	-7.79	-1.71	14.55	11.98	1.00	-1.10	-0.62	-0.08	1.40	0.77	0.04
55	1.412	0.200	0.282	3.11	3.95	2.76	1.96	2.27	1.29	1.76	2.06	0.88	-10.23	-12.78	-2.81	11.38	14.74	3.40	-0.97	-1.01	-0.25	1.14	0.88	0.26
56	1.441	0.196	0.282	3.22	4.28	2.49	2.05	2.65	1.59	1.93	2.25	0.83	-12.82	-10.47	-3.70	8.75	12.87	1.74	-0.91	-0.87	-0.21	0.98	0.93	0.22
57	1.516	0.212	0.321	3.27	3.69	3.06	1.84	2.12	1.99	2.04	1.78	1.16	-12.48	-9.45	-2.14	9.13	9.96	4.63	-1.14	-0.82	-0.20	1.02	0.84	0.44
58	1.571	0.192	0.302	3.28	3.30	2.77	2.14	2.12	1.69	1.84	1.76	1.63	-12.78	-8.97	-1.99	9.77	9.31	4.23	-1.06	-0.71	-0.11	1.02	0.93	0.51
59	1.702	0.180	0.306	3.64	4.42	2.82	2.49	2.44	1.98	2.15	2.41	0.89	-15.04	-12.87	-3.68	12.65	14.73	3.02	-1.08	-0.98	-0.28	1.32	1.18	0.25
60	1.824	0.160	0.292	3.39	4.27	3.18	1.94	2.37	2.21	2.28	2.45	1.40	-18.43	-8.65	-4.67	11.13	15.08	4.09	-0.95	-0.67	-0.18	1.20	0.92	0.35
61	1.885	0.184	0.347	4.19	4.53	2.95	2.51	2.69	2.28	2.49	2.60	1.11	-15.85	-11.04	-4.56	13.29	15.04	4.41	-1.19	-0.91	-0.36	1.43	1.08	0.40
62	1.987	0.172	0.342	4.14	3.11	3.01	2.65	2.22	1.69	2.19	1.71	1.72	-19.54	-5.33	-1.43	14.80	9.91	5.38	-1.24	-0.39	-0.07	1.42	0.56	0.60
63	2.078	0.172	0.357	3.96	4.79	3.06	2.73	2.86	2.31	2.47	1.95	0.92	-14.56	-10.09	-4.86	16.65	13.85	2.23	-1.11	-0.84	-0.21	1.47	0.82	0.21
64	2.105	0.180	0.379	4.69	4.32	3.44	2.71	2.56	2.20	2.95	2.66	1.25	-16.62	-9.42	-2.15	16.66	12.90	4.65	-1.26	-0.75	-0.17	1.74	0.93	0.41
65	2.437	0.160	0.390	4.13	5.38	4.28	2.90	3.38	2.31	2.57	2.30	1.48	-22.35	-5.73	-3.53	17.01	7.30	4.80	-1.32	-0.43	-0.11	1.49	0.52	0.55
66	2.866	0.156	0.447	3.75	5.56	5.05	2.67	3.73	2.94	2.28	2.43	2.41	-21.97	-10.02	-3.11	13.00	12.09	7.79	-1.21	-0.67	-0.15	1.30	1.06	0.74
67	1.933	0.156	0.302	4.82	3.45	0.78	2.99	1.78	0.36	2.46	2.10	0.21	-22.77	-7.24	-0.16	16.14	7.69	0.42	-1.06	-0.35	-0.01	1.63	0.68	0.03
68	2.147	0.164	0.352	4.24	4.74	0.52	2.35	2.82	0.32	2.24	2.46	0.22	-17.26	-11.06	-0.44	12.48	14.82	1.18	-1.04	-0.88	0.00	1.30	1.07	0.04
69	2.166	0.18	0.390	4.11	3.20	2.06	2.59	1.79	1.17	2.47	1.84	0.97	-17.09	-8.31	-2.92	15.29	10.53	2.34	-1.23	-0.62	-0.20	1.65	0.93	0.13
70	2.223	0.168	0.373	3.98	3.81	1.59	2.32	2.28	1.09	2.07	2.12	0.80	-16.79	-10.13	-2.51	15.37	12.22	3.34	-1.13	-0.73	-0.19	1.53	0.89	0.26
71	2.291	0.156	0.357	4.12	4.18	2.53	2.12	2.28	1.28	2.08	2.05	0.98	-12.90	-8.16	-2.83	12.83	16.51	3.65	-0.87	-0.66	-0.11	1.13	0.88	0.32
72	2.332	0.172	0.401	4.39	3.73	1.95	2.74	2.03	0.95	2.38	1.64	0.42	-19.85	-8.19	-1.67	15.07	10.90	1.52	-1.09	-0.61	-0.12	1.51	0.70	0.10
73	1.757	0.180	0.316	4.28	3.56	2.29	2.64	2.56	1.12	1.97	1.90	0.83	-11.74	-12.07	-3.43	11.59	13.65	3.11	-0.83	-0.85	-0.14	1.25	1.04	0.29
74	1.875	0.208	0.390	3.28	4.09	3.28	2.60	2.99	2.11	2.08	2.06	2.17	-11.05	-8.35	-2.99	9.11	7.20	6.47	-0.82	-0.83	-0.28	1.17	0.75	0.59
75	2.072	0.160	0.331	4.18	2.62	2.63	2.39	1.49	2.16	2.21	1.43	0.90	-19.93	-4.90	-2.18	13.41	10.78	3.09	-1.20	-0.34	-0.08	1.29	0.60	0.38
76	2.105	0.180	0.379	4.51	3.76	4.22	2.82	2.40	3.21	2.22	1.98	1.59	-16.70	-15.23	-9.42	10.64	13.44	7.76	-1.14	-1.09	-0.43	1.19	0.97	0.96
77	2.122	0.176	0.373	4.53	3.11	2.84	2.75	1.99	1.08	2.39	1.71	1.56	-18.85	-5.00	-1.87	14.98	8.19	5.39	-1.35	-0.36	-0.07	1.49	0.59	0.60
78	2.201	0.160	0.352	4.56	3.84	2.20	3.23	2.51	1.32	2.43	2.17	1.11	-17.56	-8.92	-1.76	17.97	15.62	3.94	-1.25	-0.66	-0.08	1.58	0.87	0.44
79	2.303	0.184	0.424	4.14	4.31	2.48	2.94	2.59	1.66	2.28	2.41	1.10	-16.84	-13.72	-2.75	11.46	15.60	3.80	-1.06	-1.20	-0.19	1.38	1.24	0.33
80	2.375	0.176	0.418	4.08	4.26	2.54	2.55	2.55	1.52	2.50	2.27	1.55	-17.37	-7.79	-0.12	15.74	12.84	4.12	-1.32	-0.59	-0.01	1.57	0.93	0.58
81	2.379	0.188	0.447	4.29	6.50	5.73	2.57	3.80	3.71	2.42	2.28	2.74	-20.60	-15.90	-6.86	13.28	10.40	7.51	-1.30	-1.27	-0.43	1.54	1.00	0.78
82	2.387	0.168	0.401	5.20	6.46	4.57	3.22	4.31	2.29	2.67	2.44	2.20	-22.62	-7.91	-3.71	15.85	5.58	5.13	-1.43	-0.51	-0.12	1.65	0.54	0.67
83	2.430	0.184	0.447	4.60	6.06	3.21	2.97	2.51	1.70	2.50	1.76	1.20	-23.61	-7.65	-2.21	14.63	13.22	3.83	-1.28	-0.55	-0.12	1.70	0.79	0.45
84	2.635	0.148	0.390	5.49	5.44	2.98	3.03	3.26	0.83	2.86	2.10	1.20	-26.20	-6.16	-1.06	16.92	8.53	5.45	-1.25	-0.16	-0.10	1.63	0.75	0.55
85	2.775	0.172	0.477	4.33	3.96	4.52	3.16	2.63	2.66	2.65	2.11	1.40	-19.92	-17.75	-10.48	18.32	16.89	6.39	-1.51	-1.16	-0.34	1.76	1.28	0.84
86	3.138	0.156	0.490	4.88	6.94	4.90	3.03	3.74	2.89	2.75	2.20	2.53	-28.90	-10.96	-4.88	16.66	7.89	7.29	-1.36	-0.78	-0.18	1.80	0.63	0.84
87	1.552	0.176	0.273	3.39	3.41	1.93	1.85	1.93	0.71	1.72	2.07	0.33	-13.21	-7.89	-0.70	9.89	7.50	0.76	-0.85	-0.31	-0.02	0.99	0.69	0.04
88	1.986	0.180	0.357	4.80	3.34	3.75	2.78	2.29	1.53	1.82	1.66	1.36	-15.53	-12.99	-3.84	12.07	12.27	6.25	-1.17	-0.82	-0.16	1.26	0.92	0.49
89	2.136	0.160	0.342	3.62	2.93	2.67	2.33	2.18	0.80	1.93	1.85	0.62	-15.46	-5.98	-0.68	12.74	8.01	2.77	-1.04	-0.29	-0.03	1.12	0.64	0.26
90	2.267	0.160	0.363	3.70	3.20	4.53	2.08	1.66	1.86	2.24	1.90	2.45	-13.83	-6.11	-0.62	16.04	8.74	5.58	-0.99	-0.31	-0.03	1.35	0.63	0.58
91	2.267	0.172	0.390	4.48	3.09	2.33	2.59	1.79	1.75	2.49	1.91	0.59	-16.37	-5.48	-1.00	17.57	9.89	1.47	-1.31	-0.42	-0.05	1.61	0.71	0.14
92	2.352	0.152	0.357	4.61	4.81	4.12	2.87	3.34	3.01	2.59	1.64	2.10	-21.13	-4.01	-1.46	17.63	4.28	3.87	-1.35	-0.27	-0.04	1.55	0.31	0.48
93	2.523	0.168	0.424	4.47	3.14	4.04	2.46	1.80	0.46	1.57	1.72	1.42	-15.67	-5.01	-0.29	11.80	8.33	1.88	-1.05	-0.31	0.00	1.04	0.53	0.24
94	2.866	0.156	0.447	3.87	3.28	3.65	2.44	1.97	2.54	2.13	1.87	0.71	-14.00	-5.76	-4.09	14.98	10.47	2.75	-0.95	-0.32	-0.26	1.32	0.67	0.22
95	1.625	0.168	0.273	4.13	3.60	2.33	2.36	1.96	1.84	2.50	1.63	1.20	-15.95	-6.85	-3.15	13.61	8.44	3.00	-1.14	-0.57	-0.14	1.14	0.54	0.31
96	1.885	0.184	0.347	4.05	3.68	4.57	2.70	2.08	3.69	2.14	2.16	1.40	-16.86	-12.27	-9.27	13.28	14.89	5.45	-1.34	-0.93	-0.41	1.38	0.96	0.73
97	1.973	0.168	0.331	3.72	4.09	4.17	2.71	2.33	2.40	2.08	2.04	1.93	-16.93	-11.71	-3.36	13.71	13.31	5.42	-1.27	-1.01	-0.15	1.26	0.80	0.62
98	1.656	0.188	0.311	3.66	3.52	2.46	2.35	1.95	1.16	2.19	1.82	0.82	-13.15	-8.52	-2.80	12.76	11.90	2.10	-1.10	-0.82	-0.17	1.33	0.81	0.24
99	1.769	0.176	0.311	3.95	2.01	1.62	1.97	1.20	0.72	2.28	1.28	0.58	-13.68	-3.76	-1.46	14.79	6.52	1.90	-1.09	-0.35	-0.07	1.42	0.34	0.11
100	1.525	0.176	0.268	4.64	5.63	3.63	2.56	2.80	2.60	2.42	2.58	1.89	-14.18	-3.48	-0.84	14.77	4.65	1.40	-1.07	-0.24	-0.05	1.24	0.41	0.13
101	2.061	0.176	0.363	3.75	3.53	2.70	2.31	2.02	1.71	2.18	2.16	0.32	-12.76	-5.63	-1.15	14.09	10.23	1.23	-0.97	-0.38	-0.08	1.41	0.70	0.11
102	1.722	0.164	0.282	3.04	3.39	5.56	1.76	1.68	2.95	1.79	2.13	1.18	-11.38	-3.61	-0.23	11.91	10.38	2.73	-0.82	-0.26	-0.01	1.10	0.58	0.30
mean	2.35	0.16	0.38	4.42	4.15	3.16	2.72	2.41	1.95	2.40	2.01	1.44	-19.95	-8.96	-3.31	15.57	11.75	3.87	-1.20	-0.61	-0.17	1.49	0.78	0.37
SD	0.55	0.02	0.07	0.76	1.10	1.15	0.47	0.63	1.02	0.36	0.37	0.96	5.92	3.47	2.74	3.68	3.68	1.81	0.25	0.27	0.13	0.29	0.22	0.20
